# 2-[2-(Methyl­amino)­benzo­yl]-2,3,4,9-tetra­hydro-1*H*-pyrido[3,4-*b*]indol-1-one

**DOI:** 10.1107/S1600536810019409

**Published:** 2010-05-29

**Authors:** Lin Li, Yu-Tao Zhang

**Affiliations:** a Department of Chemistry, Biology and Agriculture, Anshun University, Anshun 561000, People’s Republic of China

## Abstract

The title compound, C_19_H_17_N_3_O_2_, was obtained from fruits of *Evodia Rutaecarpa*. In the solid state, the dihedral angle between the 2,3,4,9-tetra­hydro-1*H*-pyrido[3,4-*b*]indol-1-one (tetra­hydro-β-carbolinone) unit and the benzoyl ring is 61.46 (3)°. In the crystal, dimers are formed through inter­molecular N—H⋯O hydrogen-bonding inter­actions. In addition, intra­molecular N—H⋯O hydrogen bonds are also observed. C—H⋯π contacts connect the dimers, leading to the formation of a three-dimensional supra­molecular network.

## Related literature

For general background to tetra­hydro-β-carbolinone derivatives, see: Jokela & Lounasmaa (1987 ▶); Vicente *et al.* (2008 ▶); Yamada *et al.* (1986 ▶). For bond-length data, see: Allen *et al.* (1987 ▶). For a related structure, see: Wakchaure *et al.* (2009 ▶). For graph-set notation for hydrogen bonds, see: Bernstein *et al.* (1995 ▶).
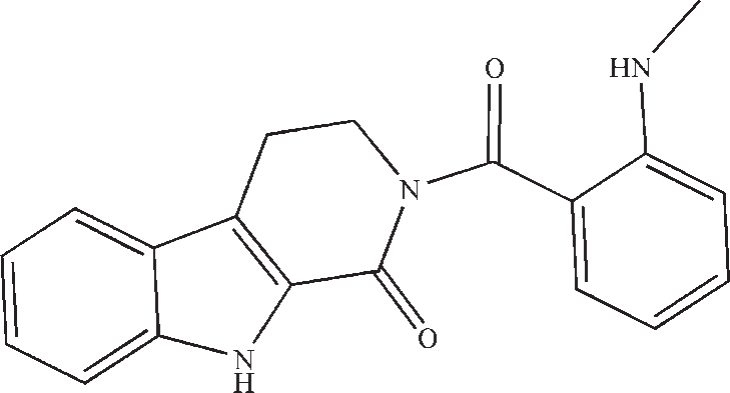


## Experimental

### 

#### Crystal data

C_19_H_17_N_3_O_2_
*M* *_r_* = 319.36
Triclinic, 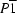

*a* = 6.9255 (14) Å
*b* = 8.8596 (18) Å
*c* = 14.158 (3) Å
α = 105.86 (3)°
β = 99.19 (3)°
γ = 96.78 (3)°
*V* = 812.9 (3) Å^3^
*Z* = 2
Mo *K*α radiation
μ = 0.09 mm^−1^
*T* = 293 K
0.28 × 0.24 × 0.19 mm

#### Data collection

Rigaku/MSC Mercury CCD diffractometer
Absorption correction: multi-scan (*REQAB*; Jacobson, 1998 ▶) *T*_min_ = 0.985, *T*_max_ = 0.996
6534 measured reflections
2927 independent reflections
1612 reflections with *I* > 2σ(*I*)
*R*_int_ = 0.051

#### Refinement

*R*[*F* ^2^ > 2σ(*F* ^2^)] = 0.059
*wR*(*F* ^2^) = 0.190
*S* = 0.94
2927 reflections
218 parameters
H-atom parameters constrained
Δρ_max_ = 0.27 e Å^−3^
Δρ_min_ = −0.21 e Å^−3^

### 

Data collection: *RAPID-AUTO* (Rigaku, 1998 ▶); cell refinement: *RAPID-AUTO*; data reduction: *CrystalStructure* (Rigaku/MSC, 2002 ▶); program(s) used to solve structure: *SHELXS97* (Sheldrick, 2008 ▶); program(s) used to refine structure: *SHELXL97* (Sheldrick, 2008 ▶); molecular graphics: *ORTEPII* (Johnson, 1976 ▶); software used to prepare material for publication: *SHELXL97*.

## Supplementary Material

Crystal structure: contains datablocks I, global. DOI: 10.1107/S1600536810019409/zl2277sup1.cif

Structure factors: contains datablocks I. DOI: 10.1107/S1600536810019409/zl2277Isup2.hkl

Additional supplementary materials:  crystallographic information; 3D view; checkCIF report

## Figures and Tables

**Table 1:** Hydrogen-bond geometry (Å, °) *Cg*1 and *Cg*2 are the centroids of the C1–C6 and C13–C18 rings, respectively.

*D*—H⋯*A*	*D*—H	H⋯*A*	*D*⋯*A*	*D*—H⋯*A*
N1—H1⋯O1^i^	0.86	2.02	2.812 (3)	153
N3—H3*A*⋯O2	0.86	2.15	2.771 (3)	129
C10A—H10*A*⋯*Cg*2^ii^	0.97	2.86	3.692 (3)	144
C19B—H19*B*⋯*Cg*1^iii^	0.96	2.88	3.586 (5)	132

Symmetry codes: (i) 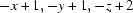; (ii) 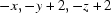; (iii) 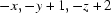.

**Fig. 1.**
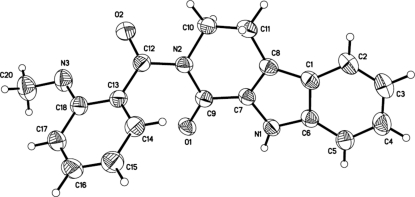
The molecular structure of the tile compound, showing 50% probability displacement ellipsoids and the atom-numbering scheme.

**Fig. 2.**
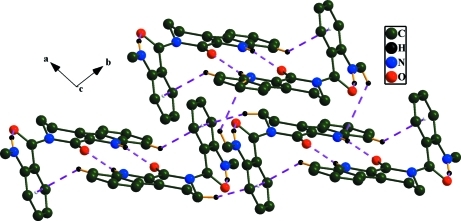
Packing diagram of the title compound; N—H···O and C—H···π interactions are shown as dashed lines. The H-atoms not involved in H-bonds have been excluded for clarity.

## supplementary crystallographic information

### Comment

The title compound, C_19_H_17_N_3_O_2_, is a tetrahydro-beta-carbolinone derivative isolated from the fruits of Evodia rutaecarpa. The tetrahydro-beta-carbolinone ring is a structural unit found in natural products. It possesses significant biological activities and plays an important role as an intermediate for the synthesis of more complex alkaloids and further functionalized polycyclic systems of biological interest (Yamada *et al.*, 1986; Jokela *et al.*, 1987; Vicente *et al.*, 2008). Herein, the conformation of the title compound has been determined by single-crystal analysis and further confirmed by mp, IR, and NMR.

As depicted in Fig. 1, the structure of the title compound, is made up of a 2-(methylamino)benzoyl ring and a tetrahydro-beta-carbolinone ring. Bond distances (Allen *et al.*, 1987) and angles are as expected and agree with the corresponding bond distances and angles reported in a related compound (Wakchaure *et al.*, 2009). The tetrahydro-beta-carbolinone ring adopts a twist conformation with atom C10 displaced by 0.547 (2) Å from the plane defined by the atoms C1-C9/C11/N1/N2. The dihedral angle between the benzoyl (C12-C18) and the tetrahydro-beta-carbolinone ring (C1-C9/C11/N1/N2) is 61.46 (3)°. An intramolecular N—H···O hydrogen bonding interactions is observed in the title compound. Two pairs of intermolecular N—H···O hydrogen bonding interactions [graph set motif is R_2_^2^(10)] lead to the formation of a dimer (Bernstein *et al.*, 1995) (Table 1). Finally, the dimers are linked into a three-dimensional supramolecular network through C—H···π stacking interactions (Fig. 2). The H-to-centroid distances of H11A···Cg1^i^ = 3.668 (2), H10A···Cg2^ii^ = 2.865 (3), H5···Cg2^iii^ = 3.082 (4) and H19B···Cg1^iv^ = 2.876 (2) Å [Cg1 and Cg2 are the centroids of the C1, C2, C3, C4, C5, C6 ring, and C13, C14, C15, C16, C17, C18 ring, respectively. Symmetry codes: (i)-1+x, y, z; (ii)-x, 2-y, 2-z; (iii)1-x, 1-y, 2-z; (iv)-x, 1-y, 2-y].

### Experimental

Dried and powdered fruits (10 kg) of Evodia rutaecarpa (juss.) were extracted with 80% EtOH (10000 mL) under reflux with a soxhlet extractor. After removing the solvent, the extract (2136 g) was suspended with water and partitioned with petroleum ether (333K-363K boiling range), CHCl_3_, EtOAc and n-BuOH, successively. The CHCl_3_ extract was evaporated to give 158 g of residues, which were separated on a silica gel (200-300 mesh) column by elution with CHCl_3_-MeOH (20:1 to 2:1) increasing polarity to give 30 fractions (2000 mL per fraction). The title compound (500 mg) was isolated from the fractions 20-22 (yield 0.32%). Single crystals suitable for X-ray diffraction were prepared by slow evaporation of a solution of the title compound in CHCl_3_ at room temperature. [m.p. 476-478 K; IR(KBr, cm^-1^): 3692(s), 3219(s), 3074(s), 2943(m), 1788(s), 1629(vs), 1510(m); ^1^H NMR (CDCl_3_) δ 10.00 (s, 1H), 7.62 (d, *J* = 8.0, 2H), 7.36-7.49 (m, 2H), 7.15-7.29 (m, 3H), 7.00 (d, *J* = 8.4, 1H), 6.80 (d, *J* = 8.4, 1H), 6.53 (t, *J* = 7.2, 1H), 4.20 (t, *J* = 6.4, 2H), 3.21 (t, *J* = 6.4, 2H), 2.98 (d, *J* = 4.2, 3H); ^13^C NMR (CDCl_3_) δ 175.8, 162.1, 151.1, 138.6, 134.5, 132.4, 126.2, 126.1, 124.9, 122.8, 120.7, 120.6, 116.1, 114.7, 113.1, 111.1, 47.5, 29.8, 21.2].

### Refinement

All H atoms were located on the difference maps, and were treated as riding atoms with C/N—H distances of 0.93, 0.96, 0.97 and 0.86 Å, for aryl, methyl, methine and amino groups, respectively, with U_iso_(H) = 1.5U_eq_ (methyl C-atoms) and 1.2U_eq _(non-methyl C-atoms). The hightest peak is located 0.62 Å from C1 and the deepest hole is located 0.96 Å from H11B.

### Crystal data

**Table:**

C_19_H_17_N_3_O_2_	*Z* = 2
*M**_r_* = 319.36	*F*(000) = 336
Triclinic, *P*1	*D*_x_ = 1.305 Mg m^−^^3^
Hall symbol: -P 1	Mo *K*α radiation, λ = 0.71073 Å
*a* = 6.9255 (14) Å	Cell parameters from 2895 reflections
*b* = 8.8596 (18) Å	θ = 2.4–27.9°
*c* = 14.158 (3) Å	µ = 0.09 mm^−^^1^
α = 105.86 (3)°	*T* = 293 K
β = 99.19 (3)°	Block, colorless
γ = 96.78 (3)°	0.28 × 0.24 × 0.19 mm
*V* = 812.9 (3) Å^3^	

### Data collection

**Table:**

Rigaku/MSC Mercury CCD diffractometer	2927 independent reflections
Radiation source: fine-focus sealed tube	1612 reflections with *I* > 2σ(*I*)
graphite	*R*_int_ = 0.051
ω scans	θ_max_ = 25.2°, θ_min_ = 3.0°
Absorption correction: multi-scan (REQAB; Jacobson, 1998)	*h* = −8→8
*T*_min_ = 0.985, *T*_max_ = 0.996	*k* = −10→10
6534 measured reflections	*l* = −16→16

### Refinement

**Table:**

Refinement on *F*^2^	Primary atom site location: structure-invariant direct methods
Least-squares matrix: full	Secondary atom site location: difference Fourier map
*R*[*F*^2^ > 2σ(*F*^2^)] = 0.059	Hydrogen site location: inferred from neighbouring sites
*wR*(*F*^2^) = 0.190	H-atom parameters constrained
*S* = 0.94	*w* = 1/[σ^2^(*F*_o_^2^) + (0.1107*P*)^2^] where *P* = (*F*_o_^2^ + 2*F*_c_^2^)/3
2927 reflections	(Δ/σ)_max_ < 0.001
218 parameters	Δρ_max_ = 0.27 e Å^−^^3^
0 restraints	Δρ_min_ = −0.21 e Å^−^^3^

### Special details

**Table:**

Geometry. All esds (except the esd in the dihedral angle between two l.s. planes) are estimated using the full covariance matrix. The cell esds are taken into account individually in the estimation of esds in distances, angles and torsion angles; correlations between esds in cell parameters are only used when they are defined by crystal symmetry. An approximate (isotropic) treatment of cell esds is used for estimating esds involving l.s. planes.
Refinement. Refinement of F^2^ against ALL reflections. The weighted R-factor wR and goodness of fit S are based on F^2^, conventional R-factors R are based on F, with F set to zero for negative F^2^. The threshold expression of F^2^ > 2sigma(F^2^) is used only for calculating R-factors(gt) etc. and is not relevant to the choice of reflections for refinement. R-factors based on F^2^ are statistically about twice as large as those based on F, and R- factors based on ALL data will be even larger.

### Fractional atomic coordinates and isotropic or equivalent isotropic displacement parameters (Å^2^)

**Table:**

	*x*	*y*	*z*	*U*_iso_*/*U*_eq_	
C1	0.2343 (4)	0.4889 (3)	0.70272 (18)	0.0617 (7)	
C2	0.2159 (5)	0.4684 (4)	0.5997 (2)	0.0770 (9)	
H2	0.1200	0.5114	0.5666	0.092*	
C3	0.3387 (6)	0.3854 (4)	0.5487 (2)	0.0887 (10)	
H3	0.3264	0.3721	0.4804	0.106*	
C4	0.4830 (5)	0.3197 (4)	0.5969 (2)	0.0866 (10)	
H4	0.5649	0.2635	0.5598	0.104*	
C5	0.5079 (5)	0.3355 (4)	0.6977 (2)	0.0735 (8)	
H5	0.6045	0.2912	0.7294	0.088*	
C6	0.3818 (4)	0.4208 (3)	0.74992 (18)	0.0594 (7)	
C7	0.2266 (4)	0.5475 (3)	0.86524 (17)	0.0548 (6)	
C8	0.1368 (4)	0.5674 (3)	0.77794 (17)	0.0577 (7)	
C9	0.1926 (4)	0.6229 (3)	0.96382 (18)	0.0548 (6)	
C10	−0.1135 (4)	0.6735 (3)	0.86977 (18)	0.0663 (8)	
H10A	−0.2008	0.7521	0.8766	0.080*	
H10B	−0.1924	0.5710	0.8615	0.080*	
C11	−0.0254 (4)	0.6657 (3)	0.77798 (19)	0.0653 (7)	
H11A	−0.1274	0.6183	0.7180	0.078*	
H11B	0.0279	0.7722	0.7785	0.078*	
C12	0.0042 (4)	0.8140 (3)	1.05117 (19)	0.0619 (7)	
C13	0.1735 (4)	0.9081 (3)	1.13003 (18)	0.0574 (7)	
C14	0.3506 (4)	0.9590 (3)	1.1046 (2)	0.0702 (8)	
H14	0.3618	0.9271	1.0377	0.084*	
C15	0.5093 (5)	1.0544 (4)	1.1747 (3)	0.0836 (9)	
H15	0.6256	1.0878	1.1556	0.100*	
C16	0.4929 (5)	1.0999 (4)	1.2738 (3)	0.0851 (10)	
H16	0.5995	1.1644	1.3221	0.102*	
C17	0.3211 (5)	1.0511 (3)	1.3023 (2)	0.0747 (8)	
H17	0.3145	1.0812	1.3699	0.090*	
C18	0.1559 (4)	0.9570 (3)	1.2313 (2)	0.0632 (7)	
N3	−0.0158 (4)	0.9133 (3)	1.26093 (17)	0.0823 (8)	
H3A	−0.1170	0.8599	1.2155	0.099*	
C19	−0.0355 (6)	0.9518 (5)	1.3638 (2)	0.1037 (12)	
H19A	0.0567	0.9042	1.3997	0.156*	
H19B	−0.1683	0.9118	1.3673	0.156*	
H19C	−0.0081	1.0653	1.3932	0.156*	
N1	0.3747 (3)	0.4570 (2)	0.84953 (14)	0.0602 (6)	
H1	0.4487	0.4286	0.8942	0.072*	
N2	0.0427 (3)	0.7161 (2)	0.96226 (14)	0.0580 (6)	
O1	0.2882 (3)	0.6119 (2)	1.04134 (12)	0.0657 (6)	
O2	−0.1687 (3)	0.8243 (3)	1.05672 (15)	0.0893 (7)	

### Atomic displacement parameters (Å^2^)

**Table:**

	*U*^11^	*U*^22^	*U*^33^	*U*^12^	*U*^13^	*U*^23^
C1	0.0664 (17)	0.0531 (15)	0.0562 (14)	0.0014 (13)	−0.0004 (13)	0.0120 (12)
C2	0.091 (2)	0.073 (2)	0.0555 (15)	0.0042 (17)	−0.0020 (15)	0.0132 (14)
C3	0.111 (3)	0.087 (2)	0.0566 (16)	0.006 (2)	0.0125 (17)	0.0095 (16)
C4	0.093 (2)	0.079 (2)	0.0716 (18)	0.0041 (19)	0.0208 (17)	−0.0021 (16)
C5	0.075 (2)	0.0658 (18)	0.0662 (17)	0.0124 (16)	0.0087 (14)	0.0012 (14)
C6	0.0629 (17)	0.0474 (14)	0.0570 (14)	0.0029 (13)	0.0020 (12)	0.0063 (11)
C7	0.0600 (16)	0.0443 (13)	0.0543 (13)	0.0100 (12)	0.0028 (11)	0.0097 (11)
C8	0.0591 (16)	0.0496 (14)	0.0581 (14)	0.0044 (12)	−0.0005 (12)	0.0147 (11)
C9	0.0592 (16)	0.0419 (13)	0.0584 (14)	0.0057 (12)	0.0027 (12)	0.0135 (11)
C10	0.0605 (17)	0.0638 (17)	0.0670 (15)	0.0118 (14)	−0.0026 (13)	0.0152 (13)
C11	0.0675 (18)	0.0577 (16)	0.0645 (15)	0.0097 (13)	−0.0050 (13)	0.0187 (13)
C12	0.0575 (17)	0.0591 (16)	0.0663 (16)	0.0117 (14)	0.0125 (13)	0.0136 (13)
C13	0.0552 (16)	0.0473 (14)	0.0645 (15)	0.0109 (12)	0.0073 (12)	0.0103 (12)
C14	0.0646 (18)	0.0565 (17)	0.0818 (18)	0.0031 (14)	0.0148 (14)	0.0110 (14)
C15	0.065 (2)	0.067 (2)	0.103 (2)	−0.0049 (16)	0.0143 (17)	0.0084 (17)
C16	0.067 (2)	0.067 (2)	0.096 (2)	−0.0014 (16)	−0.0019 (17)	−0.0001 (17)
C17	0.076 (2)	0.0574 (18)	0.0745 (17)	0.0078 (16)	0.0021 (15)	0.0017 (14)
C18	0.0668 (18)	0.0473 (15)	0.0693 (16)	0.0097 (13)	0.0095 (14)	0.0098 (12)
N3	0.0802 (18)	0.0838 (19)	0.0691 (15)	−0.0047 (14)	0.0190 (13)	0.0058 (13)
C19	0.115 (3)	0.108 (3)	0.075 (2)	−0.003 (2)	0.032 (2)	0.0076 (19)
N1	0.0659 (14)	0.0553 (13)	0.0525 (12)	0.0156 (11)	0.0008 (10)	0.0090 (10)
N2	0.0559 (13)	0.0546 (13)	0.0583 (12)	0.0138 (10)	0.0014 (9)	0.0122 (10)
O1	0.0756 (13)	0.0635 (12)	0.0550 (10)	0.0205 (10)	−0.0005 (9)	0.0170 (9)
O2	0.0582 (13)	0.1167 (19)	0.0800 (13)	0.0174 (12)	0.0118 (10)	0.0079 (12)

### Geometric parameters (Å, °)

**Table:**

C1—C2	1.402 (4)	C11—H11A	0.9700
C1—C6	1.416 (3)	C11—H11B	0.9700
C1—C8	1.417 (4)	C12—O2	1.225 (3)
C2—C3	1.357 (5)	C12—N2	1.405 (3)
C2—H2	0.9300	C12—C13	1.470 (4)
C3—C4	1.393 (5)	C13—C14	1.391 (4)
C3—H3	0.9300	C13—C18	1.410 (3)
C4—C5	1.375 (4)	C14—C15	1.371 (4)
C4—H4	0.9300	C14—H14	0.9300
C5—C6	1.392 (4)	C15—C16	1.378 (4)
C5—H5	0.9300	C15—H15	0.9300
C6—N1	1.369 (3)	C16—C17	1.376 (5)
C7—C8	1.358 (3)	C16—H16	0.9300
C7—N1	1.383 (3)	C17—C18	1.402 (4)
C7—C9	1.443 (3)	C17—H17	0.9300
C8—C11	1.500 (3)	C18—N3	1.371 (4)
C9—O1	1.222 (3)	N3—C19	1.435 (3)
C9—N2	1.402 (3)	N3—H3A	0.8600
C10—N2	1.486 (3)	C19—H19A	0.9600
C10—C11	1.510 (4)	C19—H19B	0.9600
C10—H10A	0.9700	C19—H19C	0.9600
C10—H10B	0.9700	N1—H1	0.8600
			
C2—C1—C6	118.2 (3)	H11A—C11—H11B	108.3
C2—C1—C8	134.9 (2)	O2—C12—N2	118.4 (2)
C6—C1—C8	106.9 (2)	O2—C12—C13	123.1 (2)
C3—C2—C1	119.7 (3)	N2—C12—C13	118.4 (2)
C3—C2—H2	120.2	C14—C13—C18	118.9 (3)
C1—C2—H2	120.2	C14—C13—C12	120.0 (2)
C2—C3—C4	121.0 (3)	C18—C13—C12	120.9 (3)
C2—C3—H3	119.5	C15—C14—C13	122.4 (3)
C4—C3—H3	119.5	C15—C14—H14	118.8
C5—C4—C3	122.0 (3)	C13—C14—H14	118.8
C5—C4—H4	119.0	C14—C15—C16	118.6 (3)
C3—C4—H4	119.0	C14—C15—H15	120.7
C4—C5—C6	116.9 (3)	C16—C15—H15	120.7
C4—C5—H5	121.6	C17—C16—C15	120.9 (3)
C6—C5—H5	121.6	C17—C16—H16	119.5
N1—C6—C5	129.6 (2)	C15—C16—H16	119.5
N1—C6—C1	108.2 (2)	C16—C17—C18	121.1 (3)
C5—C6—C1	122.2 (2)	C16—C17—H17	119.4
C8—C7—N1	110.7 (2)	C18—C17—H17	119.4
C8—C7—C9	126.2 (2)	N3—C18—C17	120.3 (3)
N1—C7—C9	122.8 (2)	N3—C18—C13	121.7 (3)
C7—C8—C1	106.7 (2)	C17—C18—C13	118.0 (3)
C7—C8—C11	119.7 (2)	C18—N3—C19	123.4 (3)
C1—C8—C11	133.5 (2)	C18—N3—H3A	118.3
O1—C9—N2	123.0 (2)	C19—N3—H3A	118.3
O1—C9—C7	124.0 (2)	N3—C19—H19A	109.5
N2—C9—C7	113.0 (2)	N3—C19—H19B	109.5
N2—C10—C11	111.7 (2)	H19A—C19—H19B	109.5
N2—C10—H10A	109.3	N3—C19—H19C	109.5
C11—C10—H10A	109.3	H19A—C19—H19C	109.5
N2—C10—H10B	109.3	H19B—C19—H19C	109.5
C11—C10—H10B	109.3	C6—N1—C7	107.50 (19)
H10A—C10—H10B	107.9	C6—N1—H1	126.3
C8—C11—C10	109.1 (2)	C7—N1—H1	126.3
C8—C11—H11A	109.9	C9—N2—C12	121.4 (2)
C10—C11—H11A	109.9	C9—N2—C10	117.8 (2)
C8—C11—H11B	109.9	C12—N2—C10	118.2 (2)
C10—C11—H11B	109.9		

### Hydrogen-bond geometry (Å, °)

**Table:**

Cg1 and Cg2 are the centroids of the C1–C6 and C13–C18 rings, respectively.

**Table:**

*D*—H···*A*	*D*—H	H···*A*	*D*···*A*	*D*—H···*A*
N1—H1···O1^i^	0.86	2.02	2.812 (3)	153
N3—H3A···O2	0.86	2.15	2.771 (3)	129
C10A—H10A···Cg2^ii^	0.97	2.86	3.692 (3)	144
C19B—H19B···Cg1^iii^	0.96	2.88	3.586 (5)	132

Symmetry codes: (i) −*x*+1, −*y*+1, −*z*+2; (ii) −*x*, −*y*+2, −*z*+2; (iii) −*x*, −*y*+1, −*z*+2.
